# Lateral Order and Self-Organized Morphology of Diblock Copolymer Micellar Films

**DOI:** 10.3390/polym10060597

**Published:** 2018-05-29

**Authors:** Jiun-You Liou, Ya-Sen Sun

**Affiliations:** Department of Chemical and Materials Engineering, National Central University, Taoyuan 32001, Taiwan; swdyes@gmail.com

**Keywords:** block copolymer, micelle, self-assembly, thin film, GISAXS

## Abstract

We report the lateral order and self-organized morphology of diblock copolymer polystyrene-*block*-poly(2-vinylpyridine), P(S-*b*-2VP), and micelles on silicon substrates (SiO_x_/Si). These micellar films were prepared by spin coating from polymer solutions of varied concentration of polymer in toluene onto SiO_x_/Si, and were investigated with grazing-incidence small-angle X-ray scattering (GISAXS) and an atomic force microscope (AFM). With progressively increased surface coverage with increasing concentration, loosely packed spherical micelles, ribbon-like nanostructures, and a second layer of spherical micelles were obtained sequentially. Quantitative analysis and simulations of the micellar packing demonstrates that the spatial ordering of the loosely packed spherical micelles altered from short-range order to hexagonal order when the micellar coverage increased from small to moderate densities of the covered surface. At large densities, anisotropic fusion between spherical micelles caused the ribbon-like nanostructures to have a short-range spatial order; the ordering quality of the second layer was governed by the rugged surface of the underlying layer because the valleys between the ribbon-like nanostructures allowed for further deposition of spherical micelles.

## 1. Introduction

Block copolymers (BCP) can self-assemble into ordered nanodomains with dimensional tunability and morphological diversity through control of such molecular parameters as the block volume fraction, molecular weight, chemical architecture, and segregation strength [1,2,3,4,5,6]. Amphiphilic diblock copolymers (a-BCP) comprise a hydrophobic block and a hydrophilic block, which is linked with a covalent bond [7,8,9,10]. a-BCP materials have superior self-assembly in solution. Variation of solution conditions, such as solvent quality and polymer concentration, can yield ordered nanostructures in a wide range of morphologies or hierarchical assemblies, which form via kinetic control or in an equilibrium state [7,8,9,10]. When a-BCP are dissolved in a selected solvent at a small concentration, the insoluble blocks tend to aggregate, whereas the soluble blocks maintain contact with the solvent, hence generating spherical core-shell micelles in solution [7,8,9,10]. The self-assembly of block copolymer micelles in thin films has drawn much attention because of the prospective applications in fabricating microelectronic devices [11], nanostructured substrates for plasmon resonances [12,13,14,15,16,17,18], templates for fabricating nanostructures [19,20,21,22,23], data storage systems [24,25], etc. Ordered arrays of spherical micelles of controlled size and inter-micelle distance are achievable through the manipulation of molecular masses, block volume fractions, interactions between segment and segment, segment and solvent and segment and substrate, and polymer concentrations [26,27,28,29,30,31,32,33]. Although self-assembly, whereby micellar structures with lateral spatial order become generated, is spontaneous, the presence of defects inevitably makes the micelles pack with order on a short range on a substrate. To markedly increase both orientational and positional ordering of micelles, external forces, such as zone casting or solvent annealing, were implemented [34,35,36,37].

Although the ordering of cylindrical micelles directed with a perpendicular orientation in a solvent vapor has been extensively explored [34,35,36], spherical micelles present an interesting model system to investigate the self-ordering behavior in thin films as spun from solution [38,39,40]. Many researchers have investigated the self-ordering of polystyrene-*block*-poly(2vinylpyridine), P(S-*b*-2VP), micelles in thin films mainly by means of an atomic force microscope, for which dip-coating or spin-coating methods were implemented to deposit the micelles from toluene onto a substrate [26,27,28,29,30,31,32,33]. Distinct variations in micelle size, spatial order, and morphology were found with polymer concentration [26]. Krishnamoorthy et al. applied P(S-*b*-2VP) micellar films, which were prepared by spin-coating from xylenes, as templates to fabricate inorganic nanostructures [41]. The dimensions and periodicities of the nanostructures were achieved by varying the polymer concentration and solvent. The self-assembly of micelles on SiO_x_/Si as spun from *o*-xylene and its derivatives reveals a strong dependence of the center-to-center distance on the polymer concentration. No spherical micelle grew further ribbon-like nanostructures, even micelles with a large mass fraction in xylenes. The nearest inter-micelle distance was insensitive to the polymer concentration in toluene. Spin coating from a small concentration of polymer in toluene led a large density of micelles covering a substrate [41].

In that work, the morphological and structural properties of micelles were generally addressed with direct observation by means of an AFM or a scanning electron microscope (SEM) [26,27,28,29,30,31,32,33,41]. Although these techniques provide direct images, the structural information is limited to only local areas or it lacks statistical applicability to the entire film. We implemented a grazing-incidence small-angle X-ray scattering (GISAXS) technique combined with the modeling of GISAXS data to quantitatively characterize the lateral ordering and to derive structural information (such as shape, size and size distribution) about both micelles and a wetting layer underneath the micelles, as spun from *o*-xylene [40]. Not only was the structural information obtained precisely through modeling, but also the spatial ordering was obtained quantitatively. For the system of self-ordering of micelles, as spun from *o*-xylene onto SiO_x_/Si, we distinguished four distinct spatial arrangements—disordered spherical micelles, loosely packed spherical micelles with hexagonal order, ordered spherical micelles with random loosely packed densities, and closely packed spherical micelles with short-range order [40].

A comparison of the two systems reveals a remarkably different dependence of micellar spatial ordering with the polymer concentration [26,40,41]. In particular, the morphological transformation from the fusion of spherical micelles to the growing of ribbon-like nanostructures occurred at a large coverage density for micelles that were spun from toluene, but such a morphological transformation was totally absent from the system of micelles that were spun from *o*-xylene [26,40,41]. As the mechanism of the transformation from sphere to ribbon has not been thoroughly addressed, we utilized GISAXS and the modeling of scattering data with IsGISAXS software to quantitatively characterize the structural information and the spatial ordering of self-assembled micelles spun from toluene. By analyzing the self-organization of micelles in real and reciprocal space, we aimed to acquire a full understanding of the spatial ordering and the self-organized morphology of micelles on SiO_x_/Si.

## 2. Experimental Section

### 2.1. Materials

Polystyrene-*b*-poly(2-vinylpyridine), P(S-*b*-2VP), having *M*_n_^PS^ = 48,500 g/mol, *M*_n_^P2VP^ = 70,000 g/mol, and *M*_w_/*M*_n_ = 1.13, was purchased from Polymer Source, Inc. (Dorval, QC, Canada)., and was used as received without further purification. Polymer solutions of varied concentration in the range of 0.1–1.0 wt % were prepared by dissolving various amounts of P(S-*b*-2VP) powder in toluene. The polymer solutions were sonicated for 30 min in an ultrasonic bath, and were then were equilibrated for at least a day before spin-coating. By means of spin coating, various surface-coverage densities of micelles were deposited onto silicon substrates that were cleaned by immersing in a piranha solution (3:7 *v*/*v* 30% H_2_O_2_:H_2_SO_4_) at 85 °C for 40 min, and subsequently rinsed thoroughly with deionized water. Prior to morphological characterizations, the micellar films were placed in a chamber at a maintained temperature 25 °C with humidity of less than 20% until the residual solvent evaporated to dry.

### 2.2. Characterization

The structural information of P(S-*b*-2VP) micelles in toluene was acquired by small-angle X-ray scattering (SAXS) measurements (Nano-Viewer, Rigaku, Tokyo, Japan). The parameters for operating CuK_α_ X-rays of wavelength λ = 1.54 Å were 30 kV and 40 mA. The two-dimensional (2D) SAXS data were collected by PILATUS 100 K of 83.8 × 33.5 mm^2^ and each exposure duration with X-rays took 1 h. One-dimensional (1D) profiles with scattering intensity as a function of scattering vector, *q* (*q* = (4π/λ) × sinθ, with scattering angle θ) were outputted after the camera-length calibration with silver behenate. For SAXS data, their scattered intensities are proportional to the product of the form and structure factors, *P*(*q*) and *S*(*q*). When the micelle concentration is very dilute in toluene, the distance between neighboring dispersive micelles is far, and thus the interference effect that is associated with the spatial arrangement of micelles in toluene can be ignored. Accordingly, we assumed that the structure factor was unity; i.e., S(*q*) ~ 1. Since toluene is a selectively good solvent for PS chains, the micelles existed and dispersed with a core-shell structure in toluene. For the core-shell micelles, the core comprised compact P2VP chains, whereas the shell consisted of swollen PS chains. With curve fits of SAXS data using the IGOR software (version 6.0, WaveMetrics, Lake Oswego, OR, USA) [42], we quantitatively obtained structural information, including the radius of the P2VP cores, the thickness of the PS shells, and the polydispersity of the micelles. Since the P2VP chains were in a shrinkage conformation, the electron density of the P2VP shell in toluene was the same as that of the P2VP melt. In contrast, because the PS shell was preferentially swollen by toluene, the electron density of its swollen state was between the electron density of the PS melt and that of toluene. Therefore, the electron density of the P2VP in a melt state was used as a constant parameter in the curve fits, but the electron density of the swollen PS was left to be fitted by the IGOR [42]. We also calculated the aggregation number (*n*_a_) and the number density (*n*_m_) of micelles in toluene, according to the structural information of core-shell spherical micelles [24,40].

The surface morpologies of the micellar films were identified by an atomic-force microscope (AFM, SPA400 Seiko, Seiko Instruments Inc., Chiba, Japan) in the tapping mode. AFM images were recorded with scan ranges that were between 1 μm × 1 μm or 2 μm × 2 μm by aluminum-coated silicon cantilevers with *f* = 160 kHz and *k* = 7.4 N/m. The cantilevers had a length of 150 µm, a width of 26 µm, and a thickness of 300 µm. To acquire structural details of micellar films, we also implemented GISAXS measurements, in which an incident beam of X-rays illuminated thin films at a grazing angle of 0.2°. This grazing angle is an optimal angle between the critical angle of micellar films and that of substrates. The GISAXS data of scattered X-rays by each film was collected by the 2D detector with exposure duration of 20 min. One-dimensional (1D) scattering intensity was clearly observed when in-plane and out-of-plane scan cuts were imposed on the 2D GISAXS pattern to show 1D profiles. In-plane profiles were obtained with intensity scanning by varying *q*_//_ at a given *q*_⊥_ whereas out-of-plane profiles at a given *q*_//_ were obtained with a vertical slice of intensity as a function of *q*_⊥_.

### 2.3. Modeling of GISAXS Data

To acquire quantitative details of micellar films on top of SiO_x_/Si as-spun from solutions containing various mass fractions of P(S-*b*-2VP) in toluene, both line cuts and whole 2D scattering patterns were calculated by the software IsGISAXS (version 2.6, Institute des NanoScience de Paris, Paris, France) [43]. To model the form factor, we used two types of micelle geometries, truncated core-shell sphere and hemi-spheroid, with varied lengths of the long axis (Figure S1). The interference function of a hexagonal paracrystal of parameter *D* with Gaussian isotropic disorder *σ*_D_/*D* was used to describe the spatial arrangement of micelles on top of SiO_x_/Si [43,44,45,46,47]. To account for the observed intensity modulation along direction *q*_⊥_, we introduced a parameter to describe a homogeneous layer of varied thickness *L* between a substrate and micellar objects in the simulations [47]. As a result, through the modeling simulations, the structural parameters of the micelles for the thin films that were prepared with spin coating of polymer solutions of varied concentration could be calculated. In this study, the spatial ordering and structural parameters extracted from the fits include the nearest inter-micelle distance (*D*), disorder parameter (*σ*_D_/*D*), the average core radius (<*R*>), and height (<*H*>), size distribution of the core radius (*σ*_R_), the normal and lateral thickness of the PS shell (d*R* and d*H*), and thickness (*L*) of the homogenous layer at the substrate interface.

## 3. Results and Discussion

### 3.1. Surface Morphology of P(S-b-2VP) Micelles as Spun from Toluene onto SiO_x_/Si

**Figure 1:**
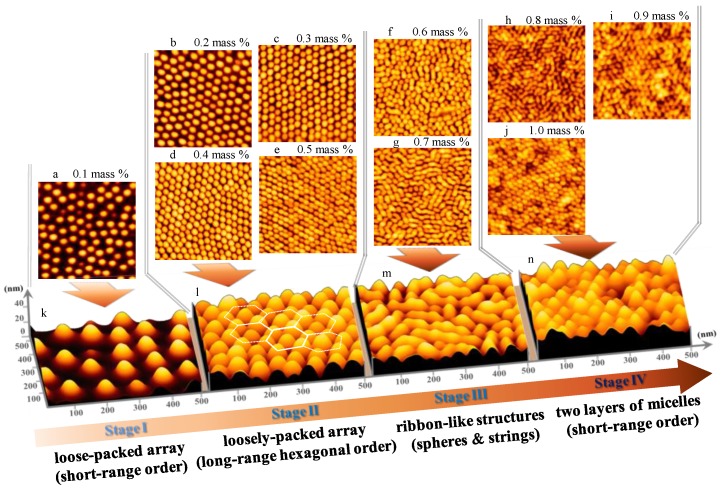
1 μm × 1 μm atomic-force microscope (AFM) topographies for micelles on SiO_x_/Si spun from toluene solutions containing polystyrene-*b*-poly(2-vinylpyridine), P(S-*b*-2VP), micelles at weight fractions: (**a**) 0.1; (**b**) 0.2; (**c**) 0.3; (**d**) 0.4; (**e**) 0.5; (**f**) 0.6; (**g**) 0.7; (**h**) 0.8; (**i**) 0.9 and (**j**) 1.0 wt %. Images (**k**–**n**), respectively, represents the three-dimensional (3D) topographies of two-dimensional (2D) images (**a**,**d**,**g**,**j**).

In the system of micelles that are spun from *o*-xylene solutions, the micellar packing and self-assembly behavior shows four spatial arrangements on SiO_x_/Si [40]. In contrast to that system [40], the micelles spun from toluene solutions reveal remarkably different packing and morphology of micelles on SiO_x_/Si, as reported by Li et al., but little interpretation was addressed to the packing and self-assembly of micelles that were spun from toluene [26]. Our intention here is to present a complete investigation of the packing and surface morphology of micelles spun from toluene by means of an AFM and GISAXS. Figure 1 shows the AFM topographies of micellar films of P(S-*b*-2VP) diblock copolymer with varied surface coverage that were prepared on cleaned silicon substrates by spin-coating (5000 rpm, 60 s) from toluene solutions containing P(S-*b*-2VP) at weight fractions in a range 0.1–1 wt %. As Figure 1 shows, various surface morphologies were obtained upon varying the surface coverage of micelles on SiO_x_/Si; the smallest density led spherical micelles to pack loosely with short-range order (0.1 wt %, Figure 1a,k). At the least surface coverage, each micelle was separate from its nearest neighbor by a large distance; one was thereby able to see clearly that the bottom of each micelle was truncated to form quasi-two-dimensional mats (Figure 1k). The collapsed micellar shells of PS chains possibly contributed to the quasi-two-dimensional mats during solvent evaporation [40]. The micelles that were obtained from toluene are smaller than the spherical micelles that were obtained from *o*-xylene [40], whereas the surface coverage of micelles that were spun from toluene is greater than that of micelles spun from *o*-xylene.

With increasing polymer concentration in the range 0.2–0.5 wt %, the spherical micelles that were packed loosely with long-range hexagonal order on SiO_x_/Si (Figure 1b–e,l). At this stage, each micelle was separate from its nearest neighbors at a preferential mean distance in a range 88–61 nm. With an increasing concentration of the polymer, ribbon-like nanostructures grew via anisotropic merging of spherical micelles (0.6–0.7 wt %, Figure 1f,g,m); two layers of micelles eventually deposited on SiO_x_/Si (0.8–1.0 wt %, Figure 1h–j,n). In the latter case, the surface morphology of the second layer was dominated by spherical micelles with short-range order. We have shown that, as spun from toluene, the micelles within the bottom layer favored merging to form ribbon-like nanostructures once they became in close contact with their neighbors at a large surface coverage. When there was no more space available for further deposition of spherical micelles at the bottom layer, certain micelles started to deposit in valleys between two ribbon-like nanostructures, yielding a second layer of micelles. Because of the rugged surface of the bottom layer, the micelles at the second layer were less likely to form ribbon-like structures via fusion. One reason is that the rugged surface of the bottom layer imposed a large barrier for the merging of the spherical micelles of the second layer as the rate of fusion depends on the probability of micellar collisions, which is proportional to the rate of relaxation of the micelles [48,49]. The spherical micelles of the second layer are expected to stick strongly to the underlying layer of ribbon-like nanostructures. This condition also increases the barrier for the merging of spherical micelles of the second layer. For both reasons, the second layer was dominated by spherical micelles rather than by ribbon-like nanostructures.

### 3.2. Experimental and Simulated GISAXS Patterns of Micellar Films on SiO_x_/Si

**Figure 2:**
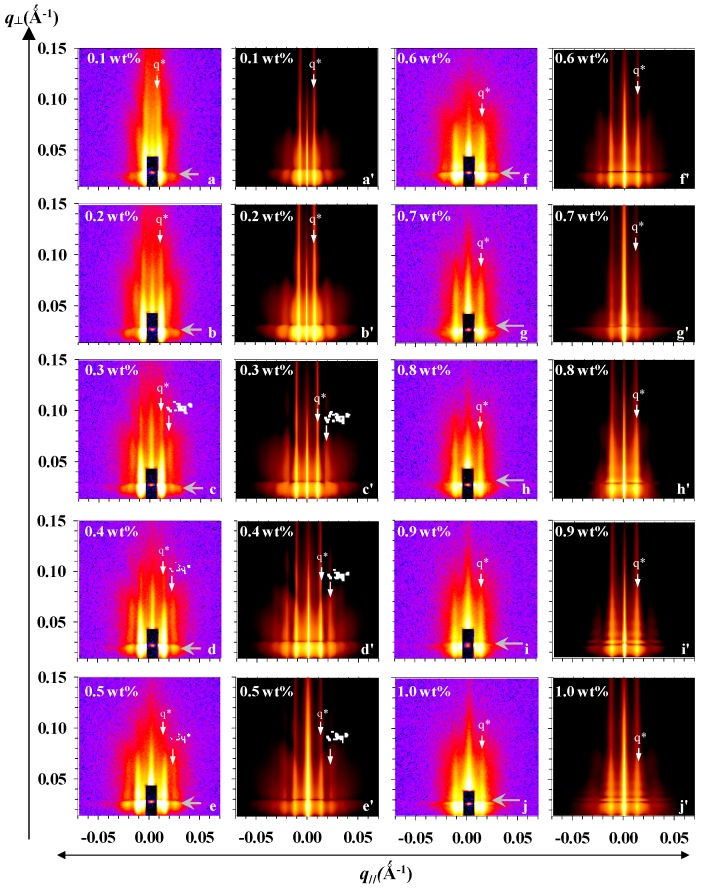
Grazing-incidence small-angle X-ray scattering (GISAXS) patterns (**a**–**j**) and IsGISAXS simulations (**a′**–**j′**) of micellar film on SiO_x_/Si spun from toluene solutions containing P(S-*b*-2VP) diblock copolymer at weight fractions: (**a**,**a′**) 0.1; (**b**,**b′**) 0.2; (**c**,**c′**) 0.3; (**d**,**d′**) 0.4; (**e**,**e′**) 0.5; (**f**,**f′**) 0.6; (**g**,**g′**) 0.7; **(h**,**h′**) 0.8; (**i**,**i′**) 0.9 and (**j**,**j′**) 1.0 wt %. All of the experimental images show a rod-shaped shadow resulted from the attenuation of a direct beam of X-rays by a copper beam stop. The intensity scale is logarithmic. Bright color (e.g., white & yellow) indicates high intensity of X-ray scattering. Vertical arrows label the positions of the diffraction Bragg’s rods, whereas parallel arrows label the intensity undulation due to the presence of the wetting layer and/or multiple layers.

We performed GISAXS measurements to characterize the structural details of the micellar films. This grazing angle is optimal between the critical angles of the polymer and substrates, so that the nanostructures on the surface of SiO_x_/Si can be probed. All of the experimental 2D GISAXS patterns of Figure 2a–j clearly show both Bragg truncation diffraction rods and parallel strikes (indicated by parallel arrows). These diffraction rods are associated with the spatial arrangement of micelles on SiO_x_/Si; the more nearly perfect that the ordering of micelles appears on SiO_x_/Si, the more highly ordered are the rods present at large *q*_//_ values (Figure 2b–e). The parallel strikes are ascribed to an intensity undulation caused by a wetting layer of P(S-*b*-2VP) chains in close contact with the surface of SiO_x_/Si, or by the presence of micelles in multiple layers. The wetting layer is due to the preferential anchoring of free P(S-*b*-2VP) chains onto SiO_x_/Si from solution [27,28,29]. No ring-banded scattering in terms of the form factor of monodispersed full spheres was present in the 2D GISAXS patterns; this absence indicates that the micelles that were obtained from toluene did not retain a fully spherical shape. The reason is that the PS block was swollen by toluene so that the outer shell comprised of swollen PS chains was still soft. The solid substrate thus caused a deformation of the micelles along the substrate normal during the spin coating [40]. The profile of micellar films at the substrate interface was smooth because of the normal truncation of micelles by SiO_x_/Si, but the micellar films had a rugged free surface with hemispherically shaped contours. The micellar films thus lacked a correlated roughness between the free surface and the substrate interface. This argument is supported by the absence of Kiessig fringes along the *q*_⊥_ direction at *q*_//_ = 0 Å^−1^ [46,47,50,51].

In addition to the Bragg diffraction rods, there existed form factor scattering associated with the structural information, such as shape, size, and size distribution, of the truncated micelles. Such form factor scattering appeared as inclined scattering rods in the 2D GISAXS patterns. As Figure 2a–e shows, the form factor scattering further undulated the intensity of the Bragg truncation rods along the *q*_⊥_ direction, but the undulation due to the overlap between the form factor scattering and Bragg truncation diffraction rods became indiscernible for micellar films that were prepared from concentrated solutions (0.6–1.0 wt %, Figure 2f–j). The reason is that the fusion from spherical micelles to ribbon-like nanostructures and the growing of the second layer of spherical micelles produced a large polydispersity of size and a disparity in the micelle shape. For the same reason, the hexagonal ordering of micelles was gradually destroyed. The interference among the scattered X-rays by these nanostructures consequently dominated the features of the 2D GISAXS patterns (Figure 2f–j).

The 2D GISAXS patterns for micellar films having ribbon-like nanostructures or a dual-layer thickness revealed a similar diffraction feature. This result indicates that the uppermost spherical micelles had a spatial order that was similar to that of the underlying layer comprised of ribbon-like nanostructures. This phenomenon further supports that the ordering of micelles of the top layer might be directed by the underlying layer through the deposition of spherical micelles onto the rugged regions between ribbon-like nanostructures.

**Figure 3:**
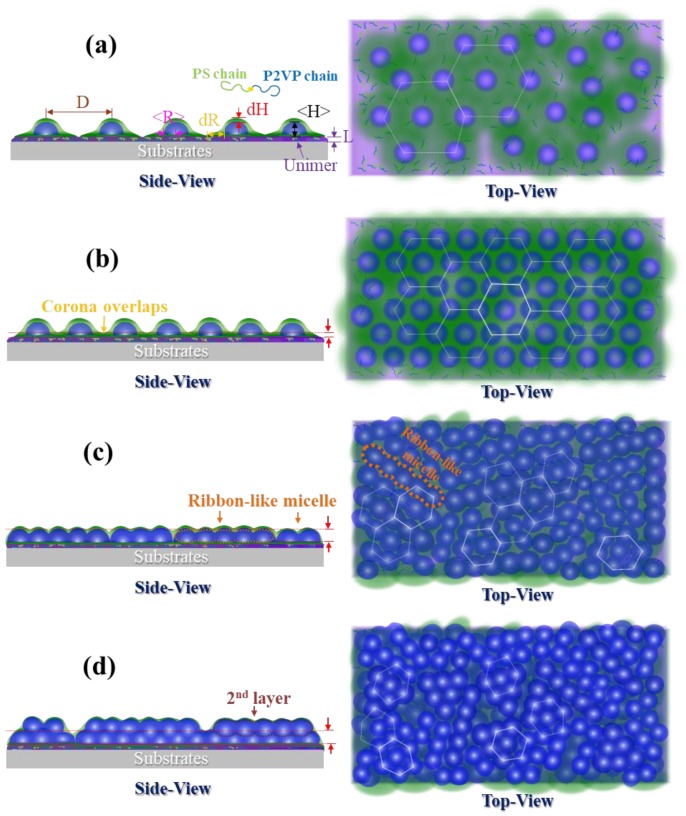
Schematic views of the four stages of P(S-*b*-2VP) micellar films: (**a**) loosely-packed spherical micelles with short-range order; (**b**) loosely packed spherical micelles with long-range hexagonal order; (**c**) ribbon-like nanostructures formed through anisotropic merging of spherical micelles; and (**d**) a second layer of spherical micelles with short-range order depositing at valleys between ribbon-like nanostructures. The radius, height of P2VP cores, the thickness of PS shells, the thickness of a homogenous layer, and the nearest-neighbor distance of spherical micelles are denoted by the labels <*R*>, <*H*>, *dR, dH*, *L*, and *D,* respectively.

To understand quantitatively the structural details of micelles on SiO_x_/Si spun from toluene, we implemented GISAXS modeling analysis based on four distinct micellar packing and morphologies. Figure 3 shows schematic illustrations of the real space of micelles with varied coverage density on top of SiO_x_/Si proposed, according to the AFM observations and GISAXS experimental data. For the first and second stages at which spherical micelles of only one type dominated the free morphology on SiO_x_/Si, we assumed that every micelle is comprised of a compact P2VP core and a layer of swollen PS shell, and that it has a truncated shape because of deformation along the substrate normal (Figure 3a,b). Because toluene evaporated rapidly, the PS shell revealed an anisotropic difference of thickness along the normal and lateral directions. We also modeled the thickness of one layer at the substrate interface because underneath the truncated micelles appeared a wetting layer of free P(S-*b*-2VP) chains that exhibited a large tendency to anchor onto the surface of SiO_x_/Si. The superimposition of mats resulting from the truncated bottom part of the micelles also contributed to the thickness of the underlying layer [40]. We used an interference function of a hexagonal paracrystal of parameter *D* with Gaussian isotropic disorder *σ*_D_/*D* to account for the spatial order of micelles and the nearest inter-micelle distance. The simulated GISAXS 2D patterns that were made on the basis of the two stages appear in Figure 2a′–e′. Only a first-order Bragg diffraction rod with sharp intensity is discernible for micelles with short-range order, whereas micelles with a hexagonal array display Bragg truncation rods in a series with sharp intensity (labeled q* and 3^1/2^ q*). 

For the third stage, increasing the surface coverage caused the growth of adjacent micelles to form ribbon-like nanostructures via fusion; these ribbon-like nanostructures and the spherical micelles consequently coexisted on top of SiO_x_/Si, but the fusion was incomplete. For the thin film that was prepared with a 0.6 wt % solution, the surface morphology was still dominated by a large population of laterally deformed spheres. Only a minor portion of short ribbon-like nanostructures formed from the merging of two or three micelles existed on the SiO_x_/Si (Figure 1f). In this case, the GISAXS pattern of such fused micelles was simulated according to the first model of truncated spheres with a large distribution of core radius (Figure 2f′).

In contrast, for the micellar film spun from a 0.7 wt % solution, the ribbon-like micelles are long, and consequently have a large aspect ratio of major to minor radius, resulting from the merging of three or more spheres. These long ribbon-like nanostructures are analogous to necklaces of deformed spheres. As this merging is a random process, the long ribbon-like nanostructures are expected to have a large polydispersity of major radius, but a narrow distribution of minor radius. To model this feature, we assumed a coexistence of two micellar shapes—a core-shell truncated sphere and a core-shell hemispheroidal shape (Figure 3c). The micellar structures of both geometries are arranged statistically with a probability 0.7 of occurrence of core-shell truncated spheres, and with a probability 0.3 of occurrence of hemispheroids. To model the micelles with a hemi-spheroid shape, we used a ratio 1.5–5 of major to minor core radius with a Gaussian distribution (Figure S1). The disorder parameter, the smallest inter-domain distance, and the thickness of the PS shell were assumed to be the same for the two geometries. The GISAXS 2D pattern simulated according to the model appears in Figure 2g′.

Because a second condensed layer appeared due to the positioning of spherical micelles in valleys within the layer of ribbon-like nanostructures, such morphology resembled a monolayer of bumps placed on a thick continuous layer. We hence modeled the GISAXS patterns based on an assumption that one layer of spherical micelles was placed on the top of a thick homogeneous layer (Figure 3d). We used the shape of truncated spheres with a core-shell structure to simulate the scattering of the layer of bumps. The simulated results for the GISAXS patterns of micelles spun from 0.8–1.0 wt % solutions are shown in Figure 2 h′–j′.

**Figure 4:**
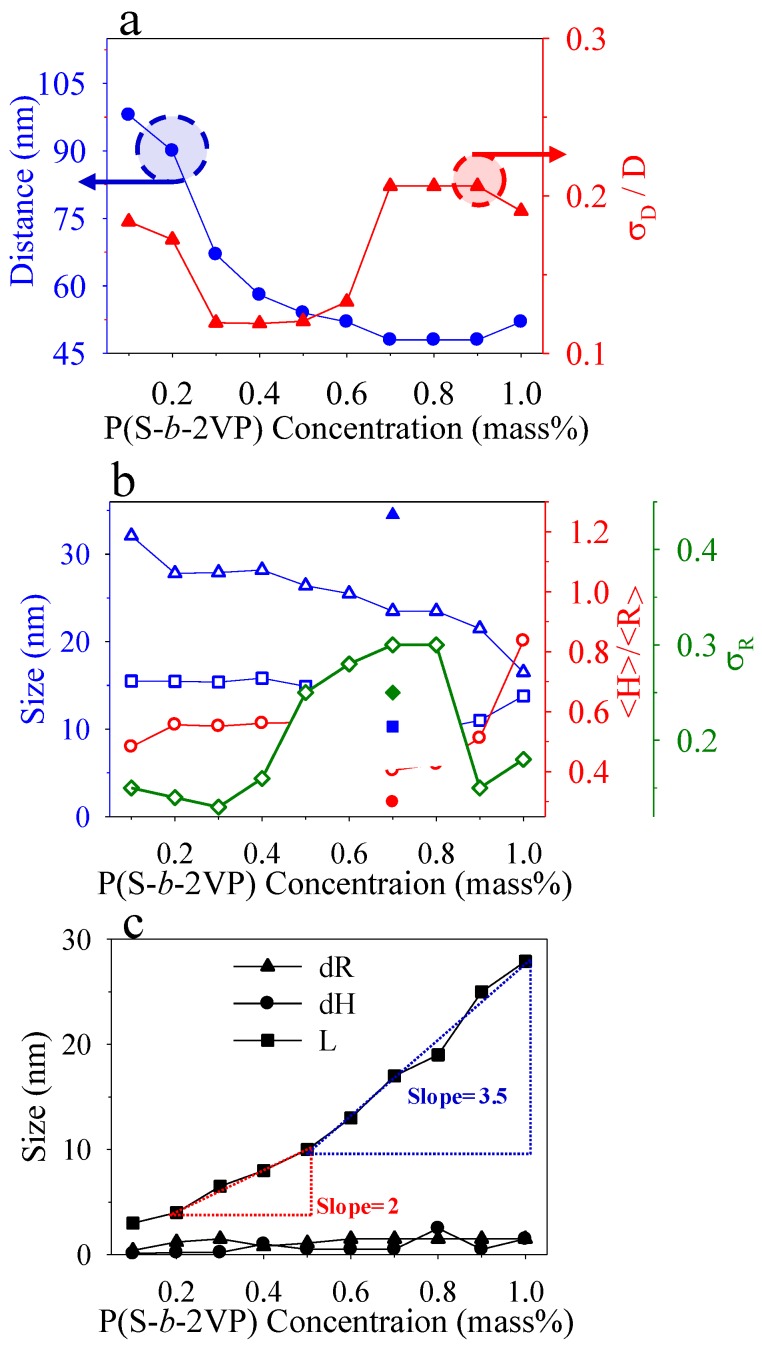
(**a**) Spatial order parameters, the nearest-neighbor distance (*D*: filled circles) and disorder parameter (*σ*_D_/*D*: filled triangles), are plotted as a function of concentrations; (**b**) Extracted heights (<*H*>: open squares), radius (<*R*>: open triangles), size distribution (*σ*_R_: open diamonds), ratio of heights to radius (<*H*>/<*R*>: open circles), of the core shape are plotted as a function of concentrations. Solid symbols with a superscript e indicates the structural parameters for hemi-spheroid cores; the ratio of major to minor radius used for the modeling is 1.5–5 with a Gaussian distribution (**c**) The thickness of PS shells (*dR:* filled triangles; *dH*: filled circles) and thickness of the homogeneous layer at the substrate interface (*L*: filled squares). The dash-lined triangles indicate slopes (nm per wt %) at each stage.

Both of the simulated 2D GISAXS patterns (Figure 2a’–j’) and the simulated 1D in-plane and out-of-plane scan profiles (Figure S2) show perfect consistency with the experimental GISAXS data. The structural parameters of the truncated micelles that were extracted from the simulated patterns and profiles are plotted in Figure 4. Figure 4a shows the dependence of spatial order parameters of micelles on concentration: whereas the inter-micelle distance *D* deceased with increased concentration, the disorder parameter *σ*_D_/*D* behaved differently. A strong decrease of *D* is observable for micelles that were spun from solutions in the range 0.1–0.5 wt %; *D* varies slightly with concentration in the range 0.6–1.0 wt %. The slight decrease of *D* with concentration in the range 0.6–1.0 wt % indicates that the micelles were in close contact with their neighbors or began to fuse to form ribbon-like nanostructures. The disorder parameter *σ*_D_/*D* is least in the range 0.3–0.5 mass%. This result indicates that the micelles spun from solutions with concentration in that range have the greatest long-range spatial order, but further increased surface coverage led to a large increase of *σ*_D_/*D*, due to the development of ribbon-like nanostructures with large polydispersity via anisotropic merging of spherical micelles and to development of the second layer of spherical micelles.

Figure 4b shows the size and the size distribution of the core structure. Whereas, the core radius and height is constant in the range 0.2–0.5 wt %, the distribution of core radius decreases with a concentration below 0.3 wt % and then increases. At the region 0.6–0.8 wt %, in which the transformation from sphere to ribbon occurred, the core radius and height both decreased with concentration. In contrast, the core radius distribution showed an opposite trend of an increase with concentration. This increase of *σ*_R_ indicates that the transformation from sphere to ribbon occurred via the random merging of spherical micelles, making micelles have a large polydispersity. At 0.7 wt %, the minor radius of hemispheroids used to describe the ribbon-like nanostructures was larger than the spherical radius, but the height of hemi-spheroids was nearly the same as the spherical radius. This condition demonstrates that the transformation from sphere to ribbon involved only an anisotropic fusion in the plane of SiO_x_/Si. After transformation was completed, further increasing concentration at 0.8–1.0 wt % caused a second layer of spherical micelles lying above the first. At this region, <*R*>, <*H*>, and *σ*_R_ reflect the radius, height, and size distribution, respectively, of the cores within the top layer. For the second layer of spherical micelles, the core radius continuously decreased with concentration, but the core height increased with concentration. The decreasing radius implies a lateral shrinkage of the second layer of micelles through the confinement imposed by the ribbon-like nanostructure underneath. Such a lateral shrinkage can relax at the free surface, thus leading to an increased height of the spherical cores at the second layer. As a result, the ratio of core height to core radius increased in the concentration range 0.8–1.0 wt %. The cores at the second layer had a small polydispersity because no transformation from sphere to ribbon occurred for the top layer.

Figure 4c shows the plots of the shell and layer thicknesses versus the concentration of the polymer. The lateral and normal thicknesses of PS shells were much smaller than for the case of micelles as spun from *o*-xylene. The thickness of the underlying layer *L* reveals two increasing behaviors. The first-step increase in *L* is discernible in the range 0.2–0.5 wt %. As shown in Figure 1a,k, the bottom of the micelles was truncated to form quasi-two-dimensional mats. Increased surface coverage density produced much superposition of the mats, accounting for the increased layer thickness at the substrate interface at the first step. The second increase of *L* is discernible in the range 0.5–1.0 wt %, which is ascribed to the transformation from the sphere to ribbon of the monolayer films and the development of dual-layer films. 

**Figure 5:**
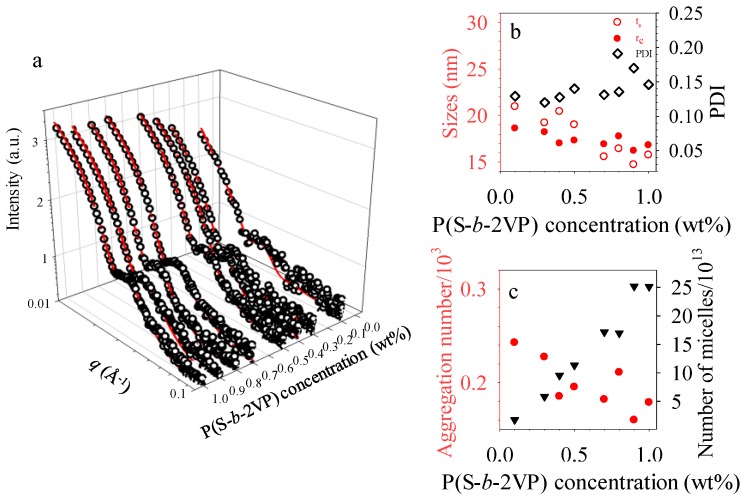
(**a**) Experimental small-angle X-ray scattering (SAXS) data (symbols) and fitted curves (lines) for P(S-*b*-2VP) core-shell micelles at varied mass fractions in toluene; (**b**) Mean radius of P2VP cores (*r*_s_, red filled circles), thickness of PS shells (*t*_s_, red open circles) and polydispersity of the P2VP cores (PDI, black open diamonds); and (**c**) aggregation number (red filled diamonds) and number of micelles (black filled triangles) as deduced from the fits are plotted as a function of P(S-*b*-2VP) concentration.

The packing and self-organized morphology of the micelles that were obtained from toluene behaved differently from those of micelles that were obtained from *o*-xylene with respect to polymer concentration. The different ordered arrangement and surface morphology of micelles in two dimensions might reflect the distinct shape, size and morphology of micelles in the various solvents. To investigate the structural details of micelles in toluene, we characterized the P(S-*b*-2VP) solutions via small-angle X-ray scattering. As Figure 5a shows, the 1D profiles reveal pronounced fringes of the form-factor scattering. These fringes are associated with structural information, such as shape, size, and size distribution of dispersed micelles in solutions [24,39,40]. Best fits were obtained with polydisperse core-shell spheres, for which the scattering length densities (SLD) of solvents and P2VP blocks were taken as ρ_toluene_ = 8 × 10^−6^ Å^−2^ and ρ_P2VP_ = 9.96 × 10^−6^ Å^−2^ for the data fitting [24]. No structural transition to form micelles of other types was induced by varying the concentration. The micelles hence retained the same core-shell spherical shape. This result indicates that the transformation from sphere to ribbon occurred in thin films during spin coating rather than in solution.

The structural parameters that were extracted from the fits are summaried in Figure 5b,c. These results indicate that each P(S-*b*-2VP) micelle in toluene comprised a hydrophilic P2VP core and a swollen hydrophobic shell of polystyrene. Toluene is an effective solvent for PS, but is poor for P2VP. The thickness of the swollen PS shell is comparable with the compact P4VP core radius, even though the molar mass of PS is less than that of P2VP. As Figure 5b shows, the micelles in toluene have a P2VP core radius 16.4–18.6 nm with a swollen PS shell of thickness 15.6–21.0 nm and a polydispersity 0.12 to 0.15. In comparison with the dimension of the core, 22.3–24.9 nm, and shell, 22.0–26.3 nm, for micelles in *o*-xylene [40], the micelles in toluene have a smaller core radius and shell thickness. As Figure 5c shows, the aggregation number *n*_a_ of micelles in toluene that was obtained from SAXS measurement was 166–242, which values are also less than those of micelles in *o*-xylene, 459–508, but the number density of micelles in toluene is approximately 2.5 times that of micelles in *o*-xylene. This larger number density explains why micelles obtained from toluene tend to form arrays on SiO_x_/Si with a large surface coverage. In addition, a comparison of the size of micelles obtained from the two solvents indicates that the micelles that were obtained from toluene have poor stability because small micelles are less energetically favored than large micelles. Furthermore, because of the thin PS shell with a small density, the activation energy for fusion is expected to be small [34]. Once the small micelles are in close contact with their nearest neighbors, such a transformation from sphere to ribbon readily occurs through fusion. As toluene evaporated quickly, the fusion was not completed so that the P2VP core slightly fused. For this reason also, ribbon-like nanostructures, rather than nanocylinders, were obtained.

## 4. Conclusions

In summary, we have demonstrated the distinct lateral ordering and self-organized morphology of P(S-*b*-2VP) micelles at varied surface-coverage densities on SiO_x_/Si, as spun from toluene. Micelles that were obtained from toluene tend to pack together to form hexagonal arrays at moderate surface-coverage densities. At large micellar coverage densities, the spherical micelles can transform to grow ribbon-like nanostructures via anisotropic fusion when they are in close contact with their nearest neighbors or deposit in valleys between ribbon-like nanostructures as a second layer. The coalescence of spherical micelles and the formation of the second layer of spherical micelles with short-range order are ascribed to the fact that the spherical micelles have a larger number density, smaller aggregation number, and smaller size in toluene than in *o*-xylene.

## Supplementary Materials

The following are available online at http://www.mdpi.com/2073-4360/10/6/597/s1. Figure S1. Schematic diagram of two micelle shapes: (a) truncated sphere and (b) hemi-spheroid. (c) Gaussian distribution of hemi-spheroid micelles.; Figure S2. Intensity cross sections of GISAXS patterns shown in Figure 2a–j (experimental data) and Figure 2a′–j′ (simulated patterns).
