# Supplementary material for: Lateral Order and Self-Organized Morphology of Diblock Copolymer Micellar Films
Source: Polymers (Basel). 2018 May 29;10(6):597. doi: 10.3390/polym10060597 (PMC6404033; doi:10.3390/polym10060597)
Supplement: Supplementary file 1 [file polymers-10-00597-s001.pdf]

Supporting Information

Lateral Order and Self-organized Morphology of Diblock Copolymer Micellar Films

Jiun-You Liou, and Ya-Sen Sun*

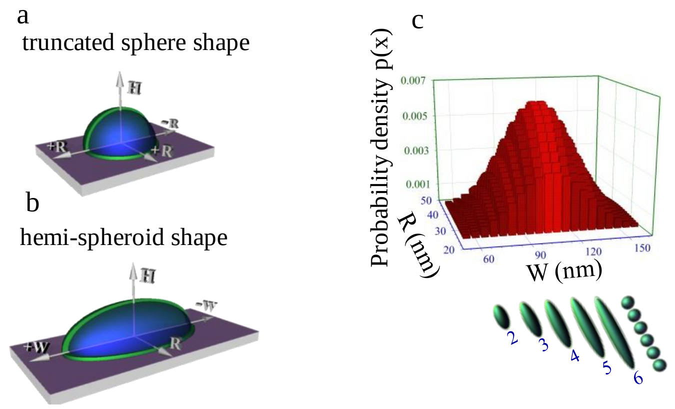

Figure S1 Schematic diagram of two micelle shapes: (a) truncated sphere and (b) hemi-spheroid. (c) Gaussian distribution of hemi-spheroid micelles. The numbers indicate the ratio of major to minor length.

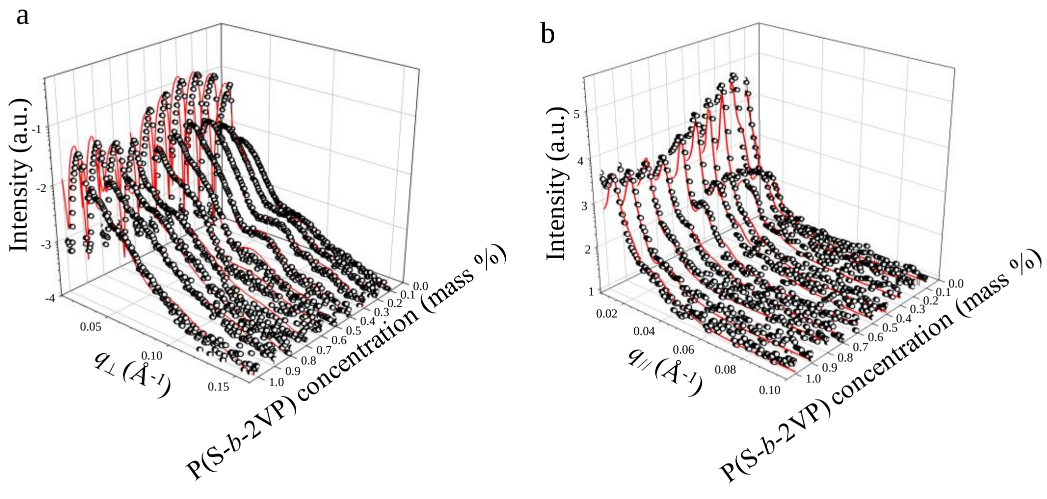

Figure S2 Intensity cross sections of GISAXS patterns shown in Figures 2 a-j (experimental data) and 2 a′-j′ (simulated patterns). The cuts were made (a) along the $q_\perp$ direction at $q_{//}$=0.1 Å^{-1} and (b) along the $q_{//}$ direction at the maximum intensity of the lobes. The black open circles represent the experimental data whereas the red lines denote the simulated profiles. The intensities are shifted for clarity along the ordinate axis.
